# Use of CdZnTe Quasi-Hemispherical Detectors under the Impact of Low Temperatures and High-Gamma Radiation Fluxes

**DOI:** 10.3390/s23208378

**Published:** 2023-10-10

**Authors:** Victor Ivanov, Viktors Fjodorovs, Anatoli Loutchanski, Maksims Piskunovs, Valerijs Ivanovs

**Affiliations:** ZRF Ritec SIA, Gustava Zemgala Av. 71A, LV-1039 Riga, Latvia

**Keywords:** CdZnTe detector, quasi-hemispherical detector, high-gamma radiation flux, IR illumination, operating temperature

## Abstract

This article discusses the possibilities and limitations of CdZnTe (CZT) quasi-hemispherical detectors operating over a wide temperature range and at high-gamma radiation fluxes. The results of the negative influence of low- and high-operating temperatures, as well as high-radiation fluxes on spectrometric characteristics of CZT detectors and possible ways to eliminate performance deterioration, are presented. The impact of infrared (IR) illumination parameters, such as wavelength and irradiation intensity on the spectroscopy performance of detectors, was investigated. A correctly chosen IR illumination wavelength and intensity were shown to significantly improve the energy resolution of CZT quasi-hemispherical detectors, allowing their stable operation in high-gamma radiation fluxes and extend the operating temperature range toward low temperatures. The influences of bias voltage values and temperatures on the quasi-hemispherical CZT detectors’ operating ability at high-gamma radiation fluxes were studied.

## 1. Introduction

Currently, CdZnTe (CZT) gamma radiation detectors are widely used to complete various tasks, including safeguards related to the control of the nonproliferation of nuclear materials, nuclear spent fuel verification and nuclear waste control. A very small size, resistance to external influences, adequate detection sensitivity for gamma radiation and stability of characteristics when operating in radiation fields of various intensities and at different ambient temperatures are necessary requirements for such devices.

There are different types of CZT detectors that can be used for such tasks. These detectors exhibit different electrode configurations (coplanar grid, pixelated, Frish-ring and other types) employed for single-charge collection realization. Among them, quasi-hemispherical detectors are widely employed [1,2,3,4,5,6,7]. These detectors are relatively simple to manufacture but exhibit favorable spectrometric characteristics and do not require complex electronic circuits for their use.

In certain tasks, CZT gamma radiation detectors must be adapted to operate at extremely high-gamma radiation fluxes, with dose rates up to 100 Gy/h or even higher. However, due to the significant deterioration in the characteristics of CZT detectors associated with radiation-induced polarization of the sensitive volume under the influence of intense gamma radiation [8,9,10], the capability of CZT detectors is limited. There are various ways to reduce or eliminate the polarization effect in detectors operated at high-radiation fluxes. This can entail the use of CZT material specifically designed for the manufacture of high-flux detectors [11] and the use of increased operating voltage values [12] when possible. To stabilize the characteristics of CZT detectors operating in pulsed current mode, it has been proposed to use infrared (IR) illumination using an IR LED for eliminating polarization [13]. Attempts to use IR illumination to improve the characteristics of different types of CZT detectors have been described in [14,15,16,17]. In [18,19], it was shown that the correctly chosen intensity and wavelength of IR illumination could significantly affect the characteristics of CZT quasi-hemispherical detectors operating at high-radiation fluxes.

The operating temperature of the detector also exerts a significant impact on its performance [20,21,22,23]. Stable operation of CZT detectors over a wide temperature range is very important when using detectors under field conditions.

The results of studies on the performance of CZT detectors operating at different operating temperatures are ambiguous. In some articles, the positive effect of using lower temperatures was described, while in other articles, degradation in detector characteristics was noted when the operating temperature was lowered.

In some publications, the possibility of improving the performance of CZT detectors with slight cooling was indicated [20] as a result of leakage current reduction. In [21], results were presented showing a significant improvement in the characteristics of detectors at low temperatures when registering low-energy X-ray radiation.

In contrast, in [22,23] the loss of the performance of CZT detectors at reduced temperatures was obtained.

Measurements of the characteristics of quasi-hemispherical detectors in the temperature range from −20 °C to +40 °C show that the characteristics of some detectors notably deteriorate with decreasing temperature [24]. By cooling CZT detectors to −15 °C and lower, there is a high probability of deterioration in the characteristics of these detectors. The effect of performance degradation at low temperatures is associated with the occurrence of a polarization phenomenon similar to radiation-induced polarization.

When detectors are operated at high X-ray radiation fluxes, even a slight decrease in temperature could lead to a significant deterioration in their characteristics [25]. Conversely, a slight increase in temperature above room temperature could improve the performance of detectors operating in high-radiation fields, despite the increase in noise associated with an increase in leakage currents with increasing operating temperature.

It was previously shown that the use of IR illumination positively affects the characteristics of quasi-hemispherical detectors when operating at low temperatures [18]. IR illumination is a low-energy consumption means being able to stabilize the characteristics of detectors at low temperatures.

In this work, the behavior of quasi-hemispherical detectors of different volumes in the operating temperature range from −40 °C to +50 °C, and at a high-gamma radiation flux with dose rate up to 0.5 Gy/h of a ^137^Cs source, was studied.

An IR illumination technique was applied to improve the performance of CZT quasi-hemispherical detectors. The influences of bias voltage values and temperatures on the quasi-hemispherical CZT detector operating ability at high-gamma radiation fluxes were studied.

## 2. Experiments and Results

For the measurements, quasi-hemispherical CZT detectors with volume of approximately 20 mm^3^, volume of 500 mm^3^, volume of approximately 1600 mm^3^ and volume of 4000 mm^3^ were fabricated.

Quasi-hemispherical detectors have a rectangular parallelepiped shape with a length–width–height ratio of A × A × (A/2). Quasi-hemispherical detectors contain a large negative electrode on five sides and a positive dot electrode at the center of one of the large sides. The optimal diameter *d* of the dot electrode is determined by the size of the detector and the electrophysical characteristics of the CZT crystal used for detector fabrication [6]. Quasi-hemispherical detectors have slightly worse spectrometric characteristics then ideal hemispherical detectors [2] due to a deterioration of charge collection from corner regions of the detector where the electric field is significantly reduced. However, the manufacturing of detectors with an ideal hemispherical geometry is a labor-consuming process. Therefore, in practice, for a simplification of the detector fabrication the quasi-hemispherical geometry is used.

CZT crystals grown by the traveling heater method (THM), produced by Redlen Technologies, were used to fabricate the detectors. All detectors were produced involving the same surface treatment. The crystals were mechanically and chemically polished. Electrochemically deposited gold contacts from a solution of 1% hydrochloroaurate acid were used. The specific resistance of the CZT crystals used for detector fabrication is >10^10^ ohm*cm, and the electron (μτ)_e_ product value is approximately 10^−2^ cm^2^/V.

**Figure 1:**
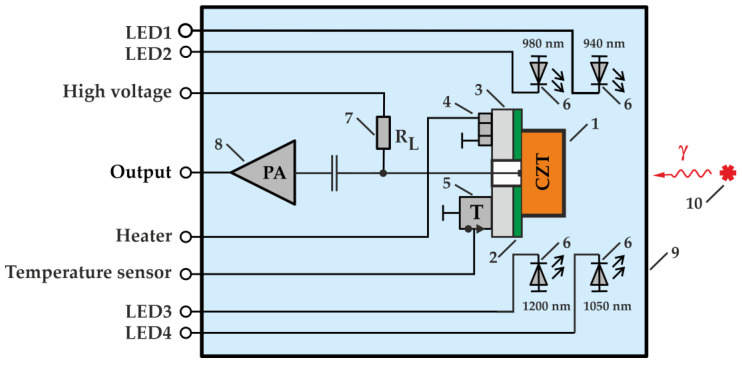
Schematic of the experimental setup: 1—CZT quasi-hemispherical detector, 2—PCB, 3—base, 4—heating element, 5—temperature sensor, 6—IR LEDs, 7—load resistor, 8—charge-sensitive preamplifier, 9—housing, 10—gamma-radiation source.

In the measurements, the CZT detectors were placed in a custom electrically shielded experimental system protected from visible light. Figure 1 shows a schematic of the experimental setup used for the measurements. A PCB (2) with a mounted CZT detector (1) is arranged on a base (3). A small heating element (4) powered by an external power supply and temperature sensor (5) used for temperature control was mounted on the base. The heating element was used to set the detector temperature above room temperature. Signals from the detector anode were applied to the input of a charge-sensitive preamplifier (8). A positive detector bias voltage was applied to the anode through a load resistor (7). A bias voltage was applied using a high-voltage power supply, allowing the measurement of detector leakage currents. Inside an electrically shielded case (9) around the detector, four IR LEDs (6) with different wavelengths of emitted light were placed. Each of the LEDs was powered by its own power source, allowing the illumination intensity to be adjusted by changing the direct consumption current of the LED. The wavelengths of the four IR LEDs used were 890 nm, 940 nm, 1050 nm and 1200 nm. In previous research [16], it was shown that the illumination of detectors at these wavelengths could yield a positive result. Sequential connection of the IR LEDs and adjustment of the LED consumption current allowed us to choose the optimal wavelength and illumination intensity for a particular detector. The optimal IR illumination intensity depends on the detector operation temperature measured by a temperature sensor and can be set by an LED current control circuit. The output pulses of the charge-sensitive preamplifier were fed to the input of a multichannel analyzer MCA527.

The experimental setup can be placed on a measuring stand in a high-gamma radiation flux for high-count-rate measurements or in a SANWOOD SM-22-CC temperature test chamber for temperature measurements in the range of −40 °C to +50 °C. In addition, the experimental setup can be mounted on the cooled side of a miniature cell phone cooler fan for a slight drop in the operating temperature for measurements without temperature chamber use. For the high-radiation flux measurements, a cell phone cooler fan available on the market was modified and used to reduce the operating temperature of the detector module by approximately 10 °C from room temperature.

### 2.1. Temperature Measurements

Measurements of the temperature-dependent characteristics of the detectors were conducted using a temperature chamber that facilitates measurements in the temperature range starting from −40 °C. To study the temperature-dependent characteristics of quasi-hemispherical detectors, detectors with volumes of 20 mm^3^, 500 mm^3^, 1600 mm^3^ and 4000 mm^3^ were used.

**Figure 2:**
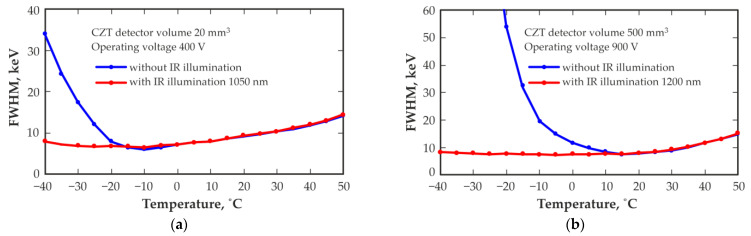
Temperature dependence of the energy resolution (FWHM) at 662 keV measured with quasi-hemispherical detectors with volumes of 20 mm^3^ (**a**) and 500 mm^3^ (**b**) without and with IR illumination.

Figure 2 shows the temperature dependence of the full width at half magnitude (FWHM) energy resolution at 662 keV of two typical detectors with volumes of 20 mm^3^ and 500 mm^3^. The energy resolution of both detectors deteriorates with increasing operating temperature above room temperature, which is associated with an increase in leakage currents leading to an increase in current noise. As the operating temperature is decreased below room temperature, the energy resolution of both detectors first slightly improves and then begins to deteriorate. The slight improvement in the energy resolution of the detectors with decreasing temperature is associated with a decrease in the current noise of the detector. The subsequent deterioration in the energy resolution may be associated with an increase in the probability of charge carrier capture at deep levels, leading to the formation of space charges and distortion of the electric field distribution in the detector. The temperature level is shown below, where notable energy resolution degradation occurs and the degradation degree vary from detector to detector; moreover, it may differ for detectors of the same size but manufactured from different CZT crystals. This may indicate a difference in the structure of deep levels and/or the presence of defects in the CZT crystals used for detector fabrication.

**Figure 3:**
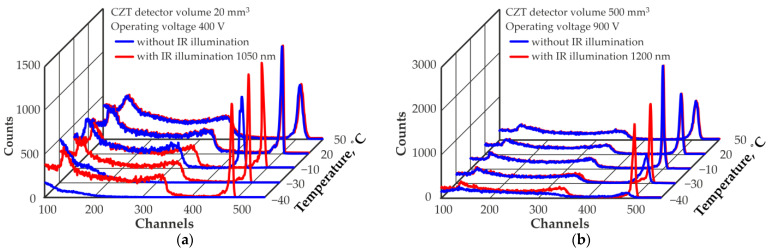
Spectra of ^137^Cs measured by a quasi-hemispherical detector with volumes of 20 mm^3^ (**a**) and 500 mm^3^ (**b**) at different operating temperatures without and with IR illumination.

Deterioration in the energy resolution with decreasing temperature can be accompanied by distortion in the shape of the recorded spectra, up to the emergence of false peaks in the spectrum. Figure 3 shows the shapes of the recorded spectra of ^137^Cs measured at different temperatures. A significant degradation in the recorded spectra up to almost complete disappearance was obtained.

The most direct way to ensure the operability of a given detector at low temperature is instrumental heating. However, this method is relatively energy consuming and requires the use of a temperature stabilization system to ensure heating only at low temperatures. We propose the use of IR LEDs to ensure the operability of detectors at low temperatures.

The use of IR illumination significantly affects the performance of these detectors, especially at low temperatures. The temperature dependence of the energy resolution (FWHM) at 662 keV of these two detectors, measured at optimal levels of IR illumination, is shown in Figure 2. The optimal level of IR illumination with a selected wavelength ensures stable operation of the detector over the entire operating temperature range with the best energy resolution. The illumination level remained constant at different operating temperatures. Figure 3 shows the influence of the optimal IR illumination level on the shapes of the recorded ^137^Cs spectra. It should be noted that at higher temperatures, the effect of the IR illumination level used is nonsignificant. The shapes of the recorded spectra with and without IR illumination are almost the same at temperatures of +20 °C and +50 °C for the 20 mm^3^ detector illuminated by IR light with a wavelength of 1050 nm, and at temperatures of −10 °C, +20 °C and +50 °C for the 500 mm^3^ detector illuminated by IR light with a wavelength of 1200 nm.

**Figure 4:**
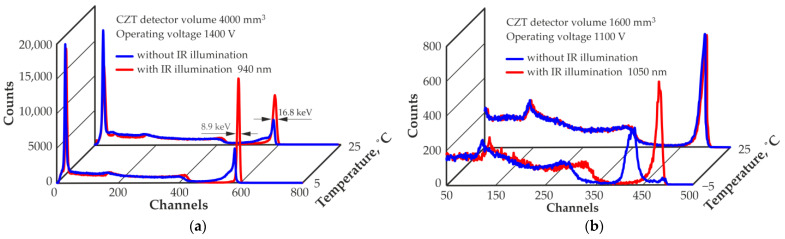
Spectra of ^137^Cs measured by a quasi-hemispherical detector with volumes of 4000 mm^3^ (**a**) and 1600 mm^3^ (**b**) at different operating temperatures without and with IR illumination.

The degree of improvement in the characteristics of CZT quasi-hemispherical detectors operating at different temperatures under the influence of IR illumination varies and is determined by the properties of the source CZT material used for detector manufacture. For example, the energy resolution of the detector with a volume of 4000 mm^3^ under the influence of the optimal IR illumination level with a wavelength of 940 nm can be improved even at room temperature and reaches the highest value of 8.9 keV at 662 keV at a temperature of +5 °C (Figure 4a). The energy resolution of the detector with a volume of 1600 mm^3^, operating at room temperature, is not affected by the IR illumination of 1050 nm, but at a temperature of −5 °C; at optimal IR illumination level and a wavelength of 1050 nm, it is possible to obtain an energy resolution of 15 keV at 662 keV and restore the spectrum shape (Figure 4b).

**Figure 5:**
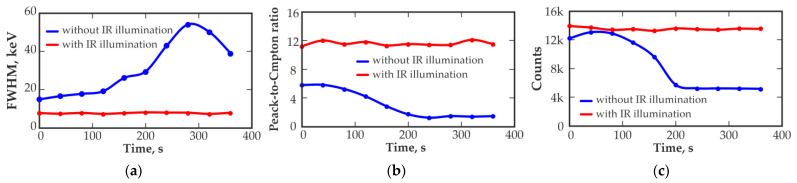
Time dependences of the energy resolution (FWHM) (**a**), peak-to-Compton ratio (**b**) and peak 662 keV area (**c**) measured with a 500 mm^3^ quasi-hemispherical CZT detector at −40 °C without and with IR illumination 1050 nm.

In addition to deterioration in the energy resolution at low temperatures, instability of the detector characteristics may appear over time. This is also due to the polarization effect in the detector. Figure 5a shows the dependence of the energy resolution (FWHM) at 662 keV of the 500 mm^3^ detector measured at −40 °C over time, Figure 5b shows the dependence of the peak-to-Compton ratio, and Figure 5c shows the dependence of the peak area at 662 keV measured under the same conditions.

**Figure 6:**
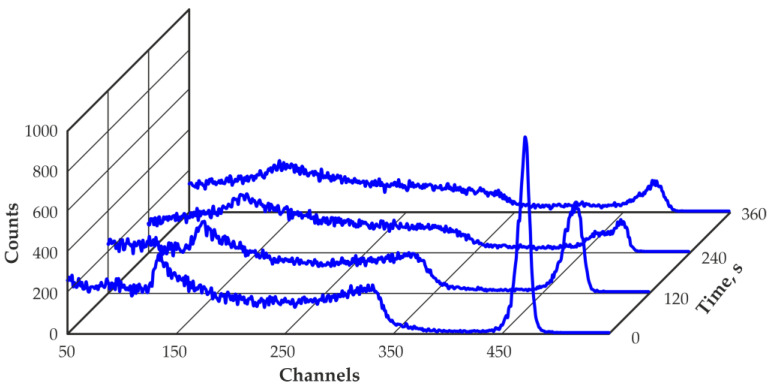
Time dependence of the ^137^Cs spectra measured with a 500 mm^3^ quasi-hemispherical CZT detector at −40 °C without IR illumination.

Deterioration in the detector performance over time can be observed. The deterioration in the energy resolution, reduction in the peak-to-Compton ratio and decrease in the registration efficiency of the total absorption peak of 662 keV are due to the formation of space charges in the detector sensitive volume, which leads to electric field distribution changes and charge collection efficiency degradation. Distortion of the shape of the recorded spectra and false peak appearance over time can be observed. The measured value of the energy resolution at the end of the measurement cycle slightly improved, but it does not indicate real improvement in the spectrometric characteristics of the detector. The time dependence of the energy resolution is accompanied by a decrease in the peak-to-Compton ratio, which is associated with a notable distortion in the shape of the recorded 662 keV peak of ^137^Cs. Indeed, Figure 6 shows the change in the shape of the measured spectra over time. A significant deterioration in the energy resolution and distortion in the shape of the recorded spectra up to several false peaks appearance can be observed.

The use of IR illumination makes it possible to stabilize the characteristics of the detector over time. Figure 5 shows the dependences of the energy resolution (a), peak-to-Compton ratio (b) and peak area (c) of the detector with IR illumination over time. Notably, there is a significant improvement and complete elimination of spectrometric parameter deterioration over time.

To achieve optimal performance over a wide operation temperature range, it is necessary to correctly select the illumination wavelength and intensity. For this purpose, the experimental measurement setup placed in the temperature chamber was used. A detector with a volume of 20 mm^3^ mounted on the PCB was placed the measurement setup. Four IR LEDs of different emission wavelengths placed near the detector were used in turn for detector illumination. The IR light illumination intensity was controlled by adjusting the direct current of the IR LEDs using an external regulated DC current source.

The measured intensity (µW/cm^2^) of IR light at the detector location was monitored using a digital optical power and energy meter type PM 100D with a remote photodiode power sensor type S122C, which allows measurements of the intensity of IR light in the wavelength range of 700–1800 nm.

**Figure 7:**
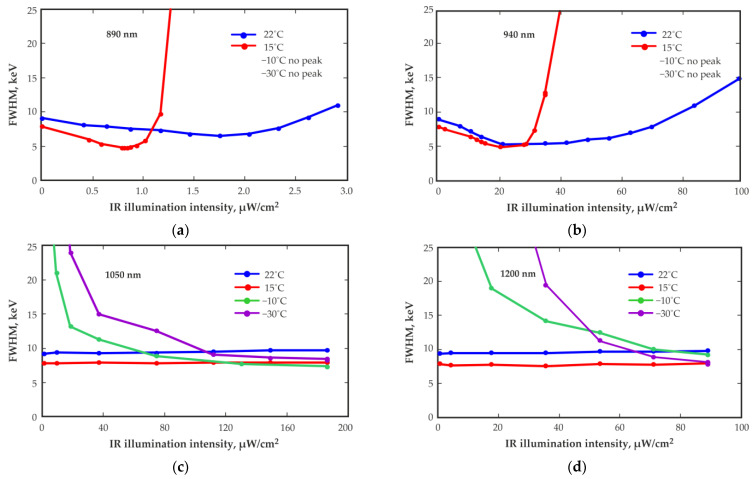
Energy resolution (FWHM) at 662 keV versus the IR light illumination intensity of various wavelengths: 890 nm (**a**), 940 nm (**b**), 1050 nm (**c**), 1200 nm (**d**), as measured with a 20 mm^3^ quasi-hemispherical CZT detector at different ambient temperatures. The detector bias voltage is 400 V.

Figure 7 shows the dependence of the energy resolution (FWHM) at 662 keV measured with a CZT detector of 20 mm^3^ on the intensity of IR light illumination of various wavelengths at different ambient temperatures. The IR LED with a wavelength of 890 nm at a direct current of 0.5 mA provides an illumination intensity at the location of the detector of approximately 3 µW/cm^2^; the IR LED with a wavelength of 940 nm at a direct current of 14 mA provides an illumination intensity of approximately 100 µW/cm^2^; the IR LED with a wavelength of 1050 nm at a direct current of 50 mA provides an illumination intensity of approximately 190 µW/cm^2^; and the IR LED with a wavelength of 1200 nm at a direct current of 50 mA provides an illumination intensity of approximately 90 µW/cm^2^. All measurements were conducted with the same bias voltage of 400 V.

Notably, it is not possible to obtain spectra at lower temperatures (below 0 °C) with IR illumination at wavelengths of 890 nm or 940 nm. At the same time, notable improvements using both mentioned wavelengths can be obtained at positive temperatures. Choosing longer-wavelength LEDs with higher illumination intensities will be needed.

In contrast, the use of longer-wavelength (1050 nm and 1200 nm) illumination makes it possible to improve the energy resolution of the detector only at lower temperatures. At lower temperatures, higher illumination levels should be applied.

The optimal illumination level is approximately the same for the IR LEDs with wavelengths of 1050 nm and 1200 nm. However, the used 1200 nm LED is less powerful than the 1050 nm LED. Therefore, the optimal illumination level of the 1200 nm LED is not reached at the maximum allowable forward current (50 mA) for this LED.

The optimal IR illumination intensity may vary for different operating temperatures and different bias voltages. It can differ from detector to detector even with the same detector dimensions and the same operating, temperature and bias voltage conditions. Among others, the correct choice of IR LED for detector illumination is important in terms of reducing power consumption. To achieve optimal illumination, it is recommended to choose an IR LED with a wavelength that provides a positive effect with minimum current consumption.

In addition, the optimal illumination intensity depends on the material of the detector electrical contacts and their thickness, the direction of IR light entering the detector, and the material and quality of processing of the inner surface of the detector housing. The latter determines the direction and amount of light reflected from the walls and other surfaces of the housing entering the detector. When IR light enters the detector through a detector surface covered by metal, the contact intensity of IR illumination should be notably increased because of light absorption by the contact material.

**Figure 8:**
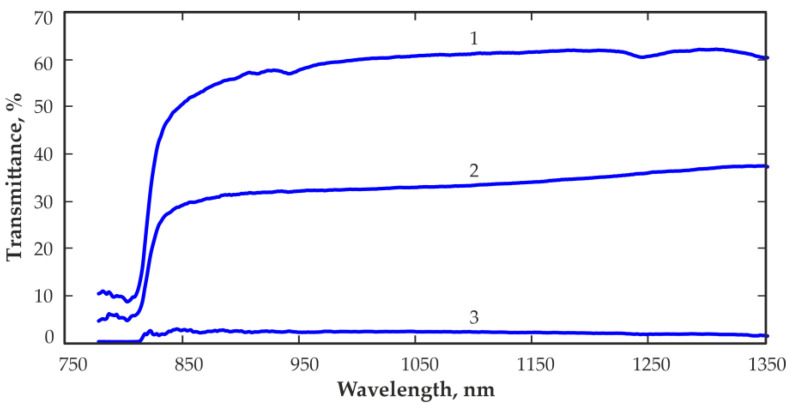
Transmittance spectra measured with a CZT sample with a thickness of 5 mm without contact (1) and with electrochemically deposited Au contacts from 0.1% (2) and 0.5% (3) hydrochloroaurate acid solutions.

Figure 8 shows transmittance spectra measured with a CZT sample with a thickness of 5 mm without any contacts (1) and with gold contacts. The gold contacts were electrochemically deposited from a solution of hydrochloroaurate acid with concentrations of 0.1% (2) and 0.5% (3). The measurements were carried out using a SARSPEC NIR spectrometer type ProNIR.

Notably, the transmittance of the CZT sample without contact is significantly higher than the transmittance of the same sample with gold contacts. The transmittance is approximately 1.7 times lower in the wavelength range of 850–1200 nm when used for contact deposition with a hydrochloroaurate acid solution concentration of 0.1%, and approximately 21–31 times lower in the same wavelength range when using a hydrochloroaurate acid solution concentration of 0.5%. The use of a more concentrated hydrochloroaurate acid solution to fabricate gold contact leads to a significantly greater reduction in light transmission by hundreds of times. Typically, at the manufacture of CZT detectors, a 1–5% hydrochloroaurate acid solution is used, which produces gold contacts with a thickness of approximately 100 nm [26]. Such a relatively thick layer of gold will significantly reduce the penetration of IR radiation into the detector if it penetrates only through the detector surfaces covered by contacts.

In most cases, it is not possible to determine one optimal IR illumination wavelength level and one optimal illumination level for a wide range of detector operating temperatures. Therefore, it is desirable to use a custom electronic scheme for adjusting the illumination intensity depending on the ambient temperature.

To ensure normal stable operation of the detectors, the highest possible bias voltage is desirable. Typically, the choice of a specific bias voltage value is determined by the level of noise associated with an increase in leakage currents with increasing bias voltage. In principle, a high-bias voltage could ensure detector operability at low temperatures. However, to ensure detector operability over a wide temperature range, it is necessary to select a bias voltage that will not lead to a significant increase in current noise at elevated temperatures. This voltage may not be optimal at lower temperatures. In this case, the use of IR illumination could ensure operation over the entire temperature range.

### 2.2. Measurements in High Gamma-Radiation Radiation Fluxes

For measurements in high-gamma radiation fluxes, equipment allowing measurements at high-count rates is needed. Detectors for measurements in strong fields must provide a relatively low registration efficiency to prevent overloading the measuring spectrometer. Small-volume CZT quasi-hemispherical detectors are suitable for this type of measurement [27,28]. However, in extremely high-gamma radiation fluxes, the performance of CZT detectors may deteriorate due to the polarization effect caused by radiation.

In our studies at high-radiation fluxes, we used quasi-hemispherical detectors with volumes of 20 mm^3^ and 5 mm^3^. To conduct the measurements, the detectors were placed in a setup similar to that for the temperature measurements. The measurement setup was placed on a certified measuring stand, which enabled measurements in the strong radiation field of the ^137^Cs source. The maximum dose rate at the measurement point was approximately 0.5 Gy/h. Changing the distance between the radiation source and the detector allows measurements in radiation fields with different dose rates.

To slightly cool the detector up to +15 °C, a cell phone cooler fan, specifically adapted for experiments, was used.

**Figure 9:**
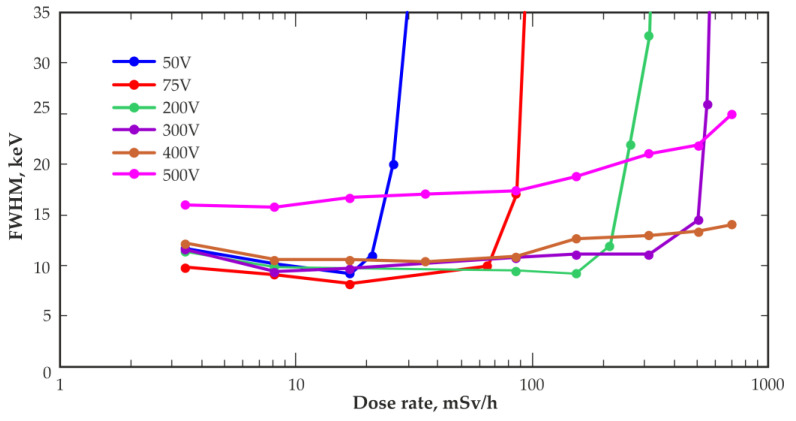
Energy resolution (FWHM) at 662 keV versus dose rate at the measurement point obtained with a 20 mm^3^ quasi-hemispherical detector at different bias voltages.

Figure 9 shows the dependence of the energy resolution (FWHM) at 662 keV measured with a typical CZT detector with a volume of 20 mm^3^ on the dose rate at the measurement points; the data were obtained at room temperature and at different bias voltages. At high-dose rates, deterioration in the energy resolution is observed up to the complete loss of detector functionality. For low-bias voltages, a deterioration in energy resolution was observed at lower dose rates. Increasing the bias voltage increased the permissible dose rate at which the detector remained operational. However, as noted above, the possibility of increasing the bias voltage of the detector is limited by the increase in leakage currents, which determines the level of current noise of the detector. The dependence of the energy resolution on the dose rate, measured at a bias voltage of 500 V, demonstrates the ability of the detector to operate at high-dose rates as well as the deterioration in the energy resolution due to the increase in current noise.

**Figure 10:**
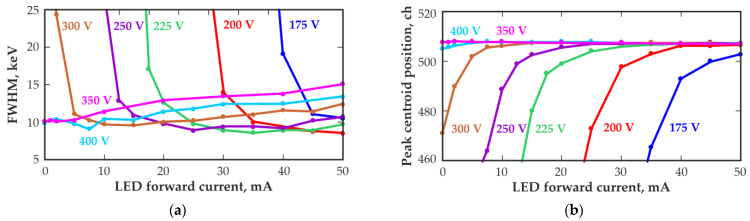
Energy resolution (FWHM) at 662 keV (**a**) and peak centroid position (**b**) versus the forward current of the 1050 nm IR LED obtained with a 20 mm^3^ quasi-hemispherical detector at different bias voltages. The dose rate at the measuring point is approximately 0.5 Gy/h.

The use of correctly selected IR illumination wavelengths and intensities could improve the performance of CZT quasi-hemispherical detectors operating in high-radiation fluxes. Figure 10 shows the dependences of the energy resolution of the detector with a volume of 20 mm^3^ and the position of the 662 keV peak centroid on the value of the IR LED forward current, which determines the illumination intensity, measured at different bias voltages at room temperature. The measurements were performed in a radiation field with a dose rate of approximately 0.5 Gy/h. Notably, using IR illumination, it is possible to ensure the same charge collection level and even a higher energy resolution at lower operating voltages than those at high-operating voltages. Moreover, to achieve optimal energy resolution at lower bias voltages, it is necessary to use a higher IR illumination intensity.

Notably, there is an optimal illumination intensity at which the best energy resolution and full charge collection can be observed for each bias voltage. It should be noted that the determined optimal illumination intensity is suitable only for a particular detector and certain measuring conditions.

**Figure 11:**
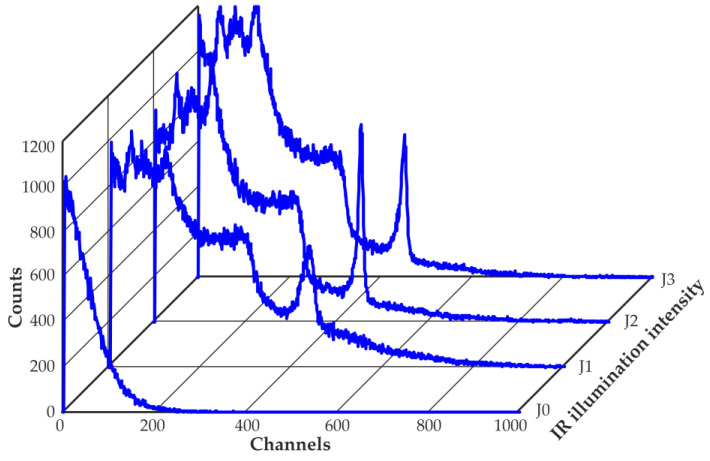
Spectra of ^137^Cs obtained with the 20 mm^3^ CZT quasi-hemispherical detector at room temperature without (J0) and with IR illumination with a wavelength of 1050 nm of different intensities J (J3 > J2 > J1 > J0). The detector bias voltage is −200 V.

Figure 11 shows the changes in the shape of the recorded spectra under the influence of IR illumination of different intensities. The ^137^Cs spectra obtained at room temperature by the CZT quasi-hemispherical detector, with a volume of 20 mm^3^ placed in a high-gamma radiation flux with a dose rate of approximately 0.5 Gy/h with and without IR illumination, of a wavelength of 1050 nm different intensities (J), are shown. During the measurements, a bias voltage of 200 V was applied. The measured count rate without IR illumination was approximately 130 kcps; with IR illumination and intensity J1, it was approximately 390 kcps; with IR illumination and intensity J2, it was approximately 410 kcps; and with IR illumination and intensity J3, it was approximately 430 kcps. The threshold in all cases was the same at approximately 40 keV.

**Figure 12:**
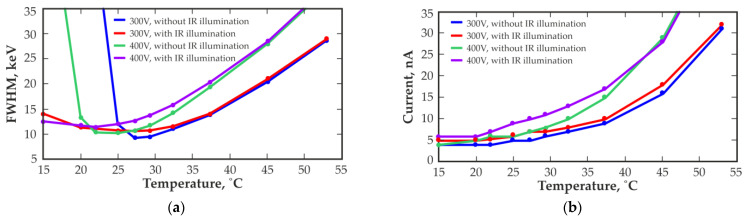
Temperature dependences of energy resolution (FWHM) at 662 keV (**a**) and detector leakage currents (**b**) measured with 20 mm^3^ quasi-hemispherical detectors at a dose rate of approximately 0.5 Gy/h at different bias voltage levels without and with IR illumination 1050 nm.

The ability to operate in high-gamma radiation fluxes is largely determined by the operating temperature. A slight change in the temperature could significantly impact the energy resolution of the detector and, in general, its operating ability. Figure 12 shows the measurement results of the energy resolution and leakage currents obtained with the detector of 20 mm^3^ in a gamma radiation field of approximately 0.5 Gy/h at different temperatures, and at bias voltages of 300 V and 400 V. Notably, even a slight decrease in the detector temperature could lead to a complete loss of detector performance. In contrast, the temperature of the detector can be increased to ensure the operability of the detector in a high-gamma radiation flux.

The use of IR illumination ensures that the detector can be operated in higher gamma radiation fluxes and at lower temperatures. The use of IR illumination at elevated temperatures imposes no effect or leads to minor degradation in the energy resolution, which is associated with an increase in leakage currents due to IR illumination.

## 3. Conclusions

When operating in high-gamma radiation fluxes and/or at low temperatures, the studied quasi-hemispherical CZT detectors showed significant dependence of the spectrometric characteristics on the bias voltage, operating temperature and intensity of the radiation field in which the measurements were performed. In addition, especially at lower temperatures, spectrometric characteristic instability could emerge. For each detector, there is a temperature below which the spectrometric properties of the detector are lost due to the occurrence of a polarization phenomenon associated with the formation of space charges and redistribution of the electric field in the detector. When operating in high-gamma radiation fluxes, the temperature at which the detector parameters deteriorate will increase. In addition, for each detector, there is a temperature value above which the energy resolution of the CZT detector significantly deteriorates relative to that at room temperature, due to an increase in leakage currents and the current noise of the detector.

Each detector exhibits a unique limit on the intensity of the gamma radiation flux, above which its functionality is lost due to the polarization effect of radiation. Polarization effects that occur at low temperatures and when exposed to strong radiation fields have the same nature, associated with the capture of charge carriers at deep levels and the formation of space charges, leading to redistribution of the electric field in the detector. Lowering the operating temperature increases the probability of the occurrence of charge carrier trapping levels. An increase in the concentration of charge carriers under the influence of gamma radiation also yields a similar effect to the above. The specific behavior of quasi-hemispherical CZT detectors at low temperatures and under the influence of a strong gamma radiation field may differ from detector to detector due to differences in the characteristics of the CZT crystals from which the detector is prepared.

When operating at room temperature in weak radiation fields, there is an optimal detector bias voltage at which operation stability, as well as the best spectrometric performance of the detector, can be achieved. A further increase in the operating voltage does not lead to improvement in the detector spectrometric characteristics. In contrast, due to the increasing leakage currents and increasing current noise, the energy resolution deteriorates. However, to realize stable operation at low temperatures and/or in high-gamma radiation fluxes, an as high as possible bias voltage is needed, which is not optimal for achieving the best spectrometric performance at room temperature and in relatively weak radiation fields.

When a further increase in the bias voltage is impossible, a fairly effective solution is to heat the detector (heating is especially favorable when the ambient temperature is below +20 °C). Thus, for example, when operating a 20 mm^3^ quasi-hemispherical CZT detector in a high-gamma radiation flux with a dose rate of 0.5 Gy/h, an increase in the detector temperature by only 5 °C (from +22 °C to +27 °C) could facilitate a reduction in the operating voltage by 25% (from 400 V to 300 V) without reducing the energy resolution, while at 22 °C and a bias voltage of 300 V, the spectrum cannot be measured (Figure 12). Conversely, when the operating temperature of the detector is reduced by 5 °C (from +22 °C to +17 °C), it is necessary to increase the operating voltage from 400 V to 550 V to achieve approximately the same energy resolution, since the performance of the detector dramatically deteriorates at +20 °C until the spectrum completely disappears at +17 °C. However, at elevated detector temperatures (over +35 °C), the dark current of the detector dramatically increases, which leads to an increase in current noise and a notable deterioration in its spectrometric characteristics.

Thus, to ensure optimal and stable operation of detectors over a wide range of operating temperatures and/or gamma-radiation fields at a relatively low-bias voltage, IR illumination can be used. In each specific case, it is necessary to determine the optimal IR illumination parameters: wavelength and intensity. Specific illumination parameters are determined by the requirements for the operating temperature range of the detector, the requirements for the maximum permissible intensity of the gamma radiation dose rates during detector use, the electrophysical characteristics of the initial CZT material used for detector manufacture, the dimensions of the detector, the material and thickness of the electrical contacts of the detector, the relative position of the detector and the IR LEDs, the dimensions and quality of processing of the detector housing inner surface, and the characteristics of the IR LEDs used.

The results obtained in this work show that to achieve detector operability under the conditions of high-gamma radiation fluxes, low temperatures and minimal bias voltages, it is necessary to use IR illumination of the detectors with a wavelength of 1050–1200 nm. For the 500 mm^3^ detector, such illumination enabled operability of the detector at the tested lower temperature limit of −40 °C and a bias voltage of 900 V, which is the optimal voltage for operation at room temperature. Applying this relatively low voltage made it possible to obtain an acceptable energy resolution of the detector at the upper temperature limit of +50 °C.

When operating at a fixed bias voltage in the widest possible range of temperatures and gamma radiation fluxes using IR illumination, it is recommended to select the detector bias voltage by determining the minimum value at which the detector can be satisfactorily operated at the lower limit of the operating temperatures, and at the maximum acceptable level of long-wavelength (≥1050 nm) IR radiation applied.

To improve the spectrometric characteristics of the large-volume quasi-hemispherical CZT detector of 4000 mm^3^ operating at positive temperatures, it is most favorable to use relatively short-wavelength IR illumination. In this particular case, illumination with wavelengths of 940 nm could be used.

The obtained results reveal that to achieve the best spectrometric characteristics of quasi-hemispherical CZT detectors operating over a wide temperature range, it is necessary to use IR illumination with different wavelengths and intensities depending on the operating temperature. A further step of this work is the development of a custom system, allowing automatic adjustments of the IR illumination light wavelength and intensity depending on the operating temperature.

## Data Availability

Not applicable.
